# An integrated oral health program for rural residential aged care facilities: a mixed methods comparative study

**DOI:** 10.1186/s12913-018-3321-5

**Published:** 2018-07-03

**Authors:** Anna Tynan, Lisa Deeth, Debra McKenzie

**Affiliations:** 1 Research Support Team, Darling Downs Hospital and Health Service, Baillie Henderson Hospital, PO Box 405, Toowoomba, QLD 4350 Australia; 2 Rural Clinical School, The University of Queensland, 152, West Street, Toowoomba, QLD 4350 Australia; 3 Tele-Health Team, Darling Downs Hospital and Health Service, Baillie Henderson Hospital, PO Box 405, Toowoomba, QLD 4350 Australia; 4 Toowoomba Oral Health Clinic, Toowoomba Hospital, Darling Downs Hospital and Health Service, 280 Pechey Street, Toowoomba, QLD 4350 Australia

**Keywords:** Oral health, Residential aged care, Tele-dentistry, Rural health

## Abstract

**Background:**

People in residential aged care facilities (RACF) are at very high risk of developing complex oral diseases and dental problems. A multidisciplinary approach incorporating oral health professionals and RACF staff is important for improving and sustaining oral health in RACFs. However, difficulties exist with access to oral health services for RACFs, particularly those in regional and rural areas. This study investigated the impact and experience of an integrated oral health program utilising tele-dentistry and Oral Health Therapists (OHT) in RACFs in a rural setting within Australia.

**Methods:**

A mixed method comparison study was undertaken. Two hundred fifty-two clinical audits were completed across nine facilities with and without access to the integrated oral health program. Twenty-seven oral health quality of life surveys were completed with eligible residents. One focus group discussions (FGD) and eight interviews were completed with RACF staff. Thematic analysis was conducted on the transcribed FGDs and IDIs. Quantitative data were analysed using descriptive statistics.

**Results:**

Audits showed an improved compliance to Australian Aged Care Quality Accreditation Standards for oral health in the facilities with access to the integrated program compared to those without the program. Thematic analysis revealed that facilities with the integrated program reported improvements in importance placed on OH, better access to OH services and training, and decreased disruption of residents, particularly those with high care needs.

**Conclusions:**

The integrated oral health program incorporating OHTs and tele-dentistry shows potential to improve the oral health outcomes of residents of RACFs. Improvements for managing oral health of residents with high care needs were observed. RACFs without easy access to an oral health service will also likely benefit from the increased support and training opportunities that the program enables.

**Electronic supplementary material:**

The online version of this article (10.1186/s12913-018-3321-5) contains supplementary material, which is available to authorized users.

## Background

Poor oral health impacts on the quality of life and general health of older people [1, 2]. It causes pain and disfigurement, and is also linked to poor nutrition, diabetes, cardiovascular disease, respiratory disease and mortality [2–9]. People in residential aged care facilities (RACFs) are at very high risk of developing complex oral diseases and dental problems [10–12]. All aged care facility residents should have regular oral health assessment and screening by trained nurses and carers however, evidence exists of high levels of oral disease and poor records of accessing Dentists within this population [11, 13]. These problems are further exacerbated by residents’ physical and cognitive impairments, medical complications, and dependence on others to maintain their oral hygiene [14–16]. Several barriers to accessing oral health services have been suggested and include: accessibility of Dentists for RACF residents (particularly in rural areas); limited or non-existent referral pathways to access dental treatment; high costs of transporting residents to a Dentist; and significant issues with moving residents from familiar surroundings to be seen at an oral health clinic (particularly residents with dementia) [17–22]. Evidence also suggests that RACF staff often lack knowledge of the specific oral hygiene requirements of older people, have limited time for adequate oral care and may also have poor attitude and awareness towards oral health [23–26]. Furthermore, having dedicated RACF staff to focus on oral health has also been met with challenges due to competing demands and appropriateness of skills [19, 27].

Access to an oral health professional in RACFs has been shown to improve oral health for residents [28, 29]. Oral health therapists (OHT) are qualified oral health professionals who are specifically trained to develop individualized oral health plans and preventative programs to reduce oral disease in the community [30]. In particular, intervention from an OHT has been shown to reduce plaque scores in residents, improve referral pathways for complex dental treatment needs and assist in establishing formal management programs for oral health within the RACFs [28]. Research has also shown that OHTs are effective in identifying RACF residents needing further treatment by a Dentist and have a positive impact on the oral health of residents who are dependent on others for oral hygiene [29, 31, 32]. However, difficulties still exist in managing residents requiring further advice and intervention from a Dentist, particularly in rural and regional areas where access issues are frequently reported.

New technologies in tele-dentistry have been developed that may assist in addressing some of the barriers for residents accessing oral health services and potentially reducing the number of referrals for face to face Dentist appointments [33, 34]. Tele-dentistry provides a viable option for remote screening, diagnosis, consultation, treatment planning and mentoring and has been successfully implemented in different settings to a variety of populations including rural, remote and other isolated populations [35–39]. Tele-dentistry is a domain of telemedicine that utilises a combination of information technology, telecommunications and dentistry for oral health consultations and education [40]. Tele-dentistry has progressed beyond live videoconferencing with a range of electronic communication technologies such as remotely monitored biometric data and the storing and forwarding of digitized data, pictures and video for non-real-time consultation [35]. In particular, live stream tele-dentistry has been developed which allows an operator to provide “live-feed” video to Dentists located offsite for further advice or review in real time [41]. Cost analysis studies of tele-dentistry has indicated no substantial difference when compared to face to face, however, access to tele-dentistry models show potential to address some of the non-economic constraints of meeting the oral health needs of aged care residents [42].

In Australia, the national oral health plan has a strong focus on improving oral health for the aged including those in RACFs [43]. In 2010 the Australian Government endorsed the Better Oral Health in Residential Care model [44]. The model promotes a multidisciplinary approach with doctors, nurses, care workers and dental professionals sharing responsibility for the four key processes of oral health screening, oral health care planning, daily oral hygiene and access to dental treatment within RACFs. Developing appropriate ways to incorporate this particularly in regional and rural areas has been an ongoing challenge. Services models incorporating tele-dentistry and OHT have emerged as a potential solution for reaching underserved and vulnerable populations including those in rural and regional RACFs [45].

An integrated oral health program incorporating an OHT and tele-dentistry has been rolled out to RACFs within a hospital and health service in Queensland, Australia. The health service includes both regional and rural populations. The program incorporates a visiting OHT for screening, education, and referral to a Dentist for a remote real-time oral examination if required. The OHT, specifically trained in manipulating the intraoral camera, can simultaneously communicate with a remotely located dentist. Facility staff and significant others are also able to participate in the session. If the tele-dentistry session indicates need for further review, an appointment at the oral health facilities is made. Details of the program have been previously described [46]. This integrated approach has never been formally examined. Previous studies on models incorporating tele-dentistry in aged care facilities have utilised different formats including use of store and forward technology completed by trained nurses [33]. The purpose of this paper is to investigate the impact and experience of this integrated approach to oral health compared to current standard care within regional and rural RACFs.

## Methods

### Study setting

The study was set in a rural health service district in Queensland, Australia. This health service covers a large geographical area (85,854 km^2^) incorporating regional and remote communities. The facilities were 6 RACFs and 3 multi-purpose health services (MPHS) that include residential aged care beds. A MPHS is generally established in populations not large enough to support a separate hospital, RACF or home and community care service. Nursing staff in these settings tend to work across a variety of hospital services including accident and emergency. These facilities have a small number of aged care beds which ranged between 4 and 5. Access to the nearest public Dentist and oral health centre for the nine facilities range from 5 km to 210kms. All facilities are governed by a single public hospital and health service.

Four of the nine facilities were engaged with the integrated oral health program and included three RACFs (with 70,40 and 14 aged care beds) and one MPHS (with 5 aged care beds). The facilities without the integrated program included three RACFs (with 80,46 and 40 aged care beds) and two MPHs (with 5 and 4 aged care beds). Not all beds were filled at the time of the study, and none of the facilities had a Dentist specifically on staff prior to the roll out of the integrated model. Residents either attended appointments at a central oral health clinic or were visited ad-hoc by a Dentist. All facilities had a designated staff member in charge of the oral health portfolio which aimed to promote oral health and lead oral health reviews.

### Study design

A mixed methods comparative study was conducted to compare aged care facilities with and without access to the integrated oral health program incorporating visiting OHTs and live-stream tele-dentistry.

### Data collection tools

#### Quantitative data collection

Quantitative data collection included an oral health related quality of life survey and a clinical audit of charts and oral hygiene setup for residents in each facility.

The audit tool was developed in 2 stages. The first stage involved development of an initial draft based on the Australian Aged Care Quality Accreditation Standard 2.15, and the National Oral Health Care Plan 2015–2024 [43, 47]. This initial draft was then circulated for review by OHTs and Dentists within the health service. Feedback and opinions were then incorporated into a new draft and recirculated for confirmation. The final audit tool comprised a review of residents’ existing oral health plan including cleaning regime, frequency of staff assessments and, mouth condition and care. A review of oral hygiene utensils such as a toothbrush, review of access to oral health professional, and documented and observed management of dentures was also included in the audit. Each OHT received training to conduct the audit and all audits were completed by one of four OHTs at each facility. The OHT initially reviewed charts and then met residents to complete review of individual needs and oral hygiene set up.

The Geriatric Oral Health Assessment Index (GOHAI) a tool specifically developed and validated for use with the elderly, was used to survey the residents [48, 49]. The GOHAI is a questionnaire about oral health quality of life and includes 12 items evaluating the three dimensions of 1) functional field (eating, speaking, and swallowing); 2) psychological field (appearance, social relationship); and 3) pain or discomfort concerning gums or teeth. Each item has a score ranging from 1 to 5 and total score ranging from 12 to 60 [49]. According to Atchison and Dolan [48], a score higher than 57 is high and indicates a satisfactory oral health quality of life. A score from 51 to 56 is average and a score of 50 or less is low, reflecting a poor oral health quality of life. Demographic data were also collected from participants on gender, highest level of education obtained, smoking habit, presence of dentures, and total time spent at the RACF.

#### Qualitative data collection

Qualitative data collection included focus group discussions (FGDs) and in-depth interviews (IDI’s) with key staff and managers at the facilities. An interview guide was developed and confirmed by the research team, piloted and adapted as necessary. Interview questions explored the experience and perceived oral health needs within the facilities, and suggested recommendations (see Additional file 1 for interview guide). The discussions were conducted by the first and second author and lasted between 45 and 60 min in duration. All interviews were digitally recorded with the consent of the participants and transcribed verbatim. De-identifiable field notes were also completed to assist with reflexivity of the experiences.

### Recruitment of study participants

Convenience sampling was used to recruit residents for the surveys with assistance from staff at each facility. Staff were asked to introduce the study to residents and to assist researchers with deciding on eligibility for the study including screening for cognitive impairment. Residents recruited for the survey all had a MMSE score > 25. All available residents’ charts and oral health equipment set up within each RACF were audited by an OHT.

Initial sampling of facility staff for discussions was completed via purposive criterion sampling whereby representatives were targeted due to their known roles or current involvement in oral health programs within the facilities. Each facility had a staff member allocated the portfolio of oral health. The primary purpose of this role was to monitor oral health needs within the facility and ensure compliance with accreditation standards. With advice from facility coordinators and managers, researchers also attended staff meetings to discuss research. Additional staff informants were recruited via snowball sampling whereby already identified respondents were asked to suggest other people who may be willing to be involved and relevant to the project.

### Analysis

Quantitative data were collected and analysed using IBM® SPSS® software version 25. The non-parametric test chi-square test of independence was used to investigate the relationship between access to the integrated oral health program and compliance with Australian Aged Care Quality Accreditation Standards for oral health. Medians, and means were also calculated for GOHAI scores. The level of significance was fixed at *p* ≤ 0.05.

Recorded discussions were transcribed verbatim and reviewed by participants to ensure accuracy. Two of the investigators independently read the transcripts to gain broad understanding of content and develop initial codes using thematic analysis. The investigators then jointly compared the themes, and re-examined the data to achieve consensus and confirm thematic saturation using a code-recode strategy. Key themes were considered around the experience of barriers and enablers to delivering an oral health program within each facility. NVivo v.11 Pro (QSL International 2015), a qualitative software data package, was used for data management.

### Ethical considerations

This study was granted ethical approval from Darling Downs Hospital and Health Service Human Research and Ethics Committee (HREC/15/QTDD/38). Written consent to participate in the study was gained from all participants. Pseudonyms have been attributed to all participants in order to ensure confidentiality.

## Results

A total of 252 audits were completed across the nine facilities and included 111 audits at facilities with the integrated oral health program and 141 audits at facilities without the integrated oral health program. Audits were included in the final data if the resident had been living at the facility for more than a month to allow time to establish an oral health plan. Not all beds were occupied at the time of the audit. Characteristics of all residents audited is outlined in Table 1.

**Table 1:** Characteristics of residents audited

	With Integrated Model	Without Integrated model
Number of aged care beds available^a^	129	171
Average Age of all residents	77.09 (34–101)	82.4 (44–97)
Gender		
Male	53 (48%)	45 (32%)
Female	58 (52%)	96 (68%)
MMSE Score^b^		
> 25	13 (12%)	33 (23%)
< 25	98 (88%)	108 (77%)

^a^ Not all facility beds were full at time of audit

^b^ A score < 25 indicated cognitive impairment on screen

Audit results showed that facilities engaged with the integrated oral health program were more likely to be implementing a satisfactory oral health plan (89.2%) compared with facilities without the integrated program (75.2%). A chi-square test revealed that this relationship was statistically significant, χ^2^ (1) = 8.037, *p* = 0.005 φ = 0.179. Results also show a statistically significant relationship with regularly replacing toothbrushes in facilities engaged with the integrated oral health program (85.6%) compared with facilities without the integrated program (68.8%) and recording last dental visit. There was no statistically significant relationship observed regarding access to a toothbrush and having a nominated Dentist recorded. There were also no statistically significant relationships observed managing residents’ dentures. Results of the audits are outlined in Table 2.

**Table 2:** Results of the oral health audit at the facilities with and without access to the integrated oral health model

RACF compliance with Best Practice: General							
	With Integrated Model (*n* = 111)		Without Integrated Model (*n* = 141)				
	N	%	N	%	χ^2^	*p*	*φ*
OH Care Plan in Place	105	94.6	122	86.5	4.526	0.033^a^	0.134
OH Care Plan Satisfactory	99	89.2	106	75.2	8.037	0.005^a^	0.179
Last Dental Visit recorded	33	29.7	17 Missing^b^ 42	17.2	4.549	0.033^a^	0.147
Nominated Dentist Recorded	78	70.3	67 Missing^b^ 44	69.1	0.035	0.851	0.013
Toothbrush available	103	92.8	132	93.6	0.067	0.796	0.16
Toothbrush regularly replaced	95	85.6	97	68.8	9.563	0.002^a^	0.196
RACF compliance with Best Practice for those with Dentures							
	With Integrated Model (those with dentures *n* = 57)		Without Integrated Model (those with dentures *n* = 95)				
	N	%	N	%	χ^2^	*p*	*φ*
Denture Cups Labelled	21	36.5	36	37.9	0.017	0.897	0.011
Denture Cups replaced weekly	26	45.6	49	51.6	0.507	0.476	0.058
Correct Storage of Dentures	30	52.63	41	43.2	1.285	0.257	0.092

^a^ Significant result

^b^ Data missing due to non-response

A total of 46 residents met the inclusion criteria to participate in a GOHAI survey. Of those 46 residents, 19 were not present at the time of the field work, or did not wish to participate in the research. Twenty-seven GOHAI surveys were completed with eligible residents. Out of the 27 participants, 7 were from a RACF with the integrated oral health program and 20 were residents from a RACF without access to the integrated oral health program. Participant characteristics of each group are shown in Table 3. The reason for the size discrepancies between the groups is due to the differences between the numbers of residents who met inclusion criteria.

**Table 3:** Demographics and GOHAI results of residents from facilities with and without access to the integrated oral health model

	With Integrated Model	Without Integrated model	Total
Gender			
Male	2 (29%)	8 (40%)	10 (37%)
Female	5 (71%)	12 (60%)	17 (63%)
Time at RACF (Average)	2.3 Years	3 years	
Smoking Habit			
Never Smoked	3 (43%)	11 (55%)	14 (52%)
Current, or former	4 (57%)	9 (45%)	13 (48%)
Education			
No formal qualification	3 (43%)	10 (50%)	13 (48%)
Completed a School Certificate	3 (43%)	7 (35%)	10 (37%)
Tertiary education or apprenticeship	1 (14%)	3 (15%)	4 (15%)
Dentures			
With	4 (57%)	16 (80%)	20 (74%)
Without	3 (43%)	4 (20%)	7 (26%)
GOHAI Scores			
Mean	50.6 ± 5.1	51 ± 5	51 ± 4.9
With High OHQoL (≥57)	0	2 (10%)	2 (7%)
With Average OHQoL (≥51- ≤ 56)	4 (57%)	10 (50%)	14 (52%)
With Poor OHQoL-(≤ 50)	3 (43%)	8 (40%)	11 (40%)

The mean GOHAI score of participants from facilities with the integrated oral health program was 50.6 ± 5.1 (median = 51; Q1 = 49; Q3 = 54.5) and participants from facilities without the integrated oral health program was 51 ± 5 (median = 52; Q1 = 47.75; Q3 = 54.25). A total of 40% of residents from RACFs without the integrated oral health program, and a total of 43% of residents from RACFs with the integrated oral health program had a score lower than 50 indicating poor oral health quality of life.

One FGD and eight IDI’s were completed with staff and managers involved in coordinating oral health portfolios at each of the facilities. Participants included 3 registered nurses (RN) and two enrolled nurse (EN) from facilities with the integrated program and 5 RNs and 3 ENs from facilities without the program. Qualitative analysis of the discussions revealed several barriers and enablers influencing the performance of managing oral health in facilities with and without access to the integrated oral health program. A summary of this is outlined in Table 4. Key themes included importance placed on oral health; access to specialist in oral health; accessing external oral health service; implications for residents with high care needs, and education and training in oral health at the facility.

**Table 4:** Barriers and enablers to oral health care at facilities with and without access to the integrated oral health model

Enablers	Barriers
RACFS without an integrated oral health service model	
⋅ Access to motivated local Oral Health Service to work with RACF ⋅ Resident access to supportive and engaged family or significant other ⋅ Formal oral health review process established in RACF	⋅ Competing priorities within RACF ⋅ No access to oral health care specialist for support and education ⋅ Time and resource intensive process for accessing oral health facilities ⋅ Management of high care resident’s oral health requires specific skills ⋅ Lack of formal oral health review process in place ⋅ Difficulties accessing family and significant other support, especially in rural areas ⋅ Poor communication between oral health facility and RACF
RACFS with an integrated oral health service model	
⋅ Promotion of preventative oral health care ⋅ Increased visibility of oral health care requirements to staff at RACF ⋅ Cost and time saving for residents, staff and RACF ⋅ Need to access dentist at an oral health facility minimised ⋅ A formal and well supported OH program established ⋅ Improved communication and follow up with oral health service ⋅ Disruption to residents especially those with dementia minimised ⋅ Model supported by OH specialist external to RACF ⋅ Increased confidence in RACF staff of managing OH needs ⋅ More opportunities for training in OH care particularly incidental training ⋅ Streamlined access to oral health appointments	⋅ Inadequate time allocated to management of OH program within RACF by dedicated staff ⋅ Delays in procurement of recommended equipment ⋅ Some RACF not well equipped to take on telehealth technology ⋅ Poorly planned and accessible telehealth facilities ⋅ Limited experience of OHT with working with residents with high needs particularly dementia

### Facilities without the integrated oral health program

#### Importance placed on oral health

For facilities without the integrated oral health program, respondents advised that in general, limited importance was placed on oral health. Respondents acknowledged that staff usually have very broad roles and therefore other priorities often took precedence.

*It is not something we go looking for. The first thing that comes out of my mouth when I start shift is ‘Did you have any problems today? Pressure ulcer? Had a fall?’ The focus for oral health is poor.* FGD Facility 1

#### Access to oral health specialist

A few of the respondents described their facility oral health program to be ad-hoc. It was believed that underlying the reason for a more ad-hoc approach to oral health was the reliance on non-specialists to drive the oral health program with many other competing demands. Concern was also noted in staff confidence in meeting the oral health needs of residents particularly those with high care needs; as one respondent explained.

*Like I said we are not specifically oral health therapists. So what we consider an issue, may not be a problem. Or something we overlook might be something that does need something.* IDI Facility 3

As a result, all respondents agreed that management of oral health needs tended to be more reactionary rather than preventative, with referral for intervention typically initiated due to observed pain, issues with eating, denture problems, or other symptoms related to residents’ mouths. However, accessing external oral health services to manage these issues was also often met with many challenges.

#### Accessing oral health service

If it was considered an emergency, most respondents thought that access to an external oral health review was typically fast. However, for any other oral health complaint, key difficulties noted included arranging oral health appointments due to need for numerous sign offs such as medical and other consents; dealing with waiting lists; and often poor communication with external oral health provider following review. In addition to this, attendance at oral health facilities relies on several additional resources from the aged care facility which incur extra costs including a staff member to attend the appointment and specialist transport such as an ambulance. For some residents with significant cognitive and mobility issues, oral health services were not always well set up to accommodate for their needs.

*Unfortunately, it is difficult for anybody who is not mobile to get over there (to access the oral health facility). If you have got someone who is bed bound, or even anyone who requires a lift or assistance with transfers to any chair. Transferring to the Dentist chair would be impossible. That is a barrier.* IDI Facility 4

#### Implications for residents with high care needs

It was felt that residents with high care needs in general were the most vulnerable to the difficulties in managing oral health in these facilities. Respondents noted that these residents also tended to have additional medical complications and other complex oral care issues such as difficulties accessing their mouths and other. The concern for these residents is highlighted by the following comment:

*It impacts worse on the ones that cannot speak for themselves, it really impacts on them because they can’t say I have a sore tooth*. FGD Facility 5

### Oral health facilitators

Despite some of the difficulties, respondents observed some key attributes which assisted in delivering an oral health program. Local access to a supportive and engaged oral health service seemed to facilitate more productive outcomes for oral health. In one case a private oral health service was on the same campus as the aged care facility. This was particularly helpful for minimising issues with transporting residents. Availability of supportive significant others also assisted with transporting issues and other difficulties noted with attending appointments. Having a well-established oral health program with a stable staff member in charge of the oral health portfolio facility was also observed as beneficial. Regular reviews also assisted with raising the profile of oral health in the facility and potentially identifying problems early.

*I think the thing that works well is when we put the oral reviews with the third monthly care plan reviews… that is done by the enrolled nurse and if there is a problem, it will trigger… well the registered nurse needs to sign off, for the registered nurse to look in their months and then we can kind off act on it in the best way we can.* FGD Facility 1

Overall, respondents advised that access to education about management of oral health was key to improving oral health of the residents. For most, it was agreed that hands-on training was likely to be more effective than online. Particularly as many voiced that one of the biggest challenges was managing residents where it was difficult to access their mouths. Respondents agreed that being shown the correct technique in this case would assist with management of these residents. Respondents advised that access to an oral health specialist for support and training would assist with this.

### Facilities with the integrated oral health program

#### Importance placed on oral health

Respondents from facilities with the integrated oral health model in place were pleased with the improved performance of managing oral health.

*Look I just think the whole thing is beneficial. It is positive for the client, it is positive for the nursing staff.* IDI Facility 7

Many suggested that the integrated model was more successful because it provided access to expert advice on the spot from the OHT on developing care plans and managing the oral health needs of the residents.

…*it is good, because everyone then gets care and everyone is checked, um you know, a specialised sort of check rather than us trying to do what we can do.* IDI Facility 8

Respondents advised that the model also meant a more formal and effective oral health program was established. With a more recognised oral health program, respondents believed that visibility of oral health as a priority in the facility became more apparent. This was particularly attributed to the visibility of the visiting OHTs to conduct the reviews and develop the care plans.

*Yes, I think it actually triggered again the importance of oral health like.... Having the girls come out here, and focused just on oral health.* IDI Facility 6

Further to this, participants also recognised that the new program had meant a more preventative approach to oral health was being implemented. Due to this most thought that that oral health issues were being attended to more promptly. This is demonstrated in the following comment.

*They* (the OHT) *can see problems that may happen in the future, like a sign of an ulcer or a sign of something. They are trained to counteract the problem or apply some preventative measure.* IDI Facility 7

#### Access to oral health specialist

The access to an oral health specialist such as the OHT to support the model was also seen as very advantageous due to their skills and specific focus in oral health. It was noted that although facility staff did try to do the best, they recognised that access to specialist skills was a great benefit particularly as more effective focus could be placed on oral health in light of competing demands within the facility. This is highlighted in the following comment.

*The biggest issues would probably be, the people who were responsible to check, are not really trained as a, you know an oral technicians… Also the time, the time factor is a major difference, we work first as nurses and then we try to fit in the time to do that as well and that depends on the staffing levels. We could have a day that might be allowed for this and then someone calls in sick and then that day would be taken because we would be needed on the floor. IDI* Facility 8

In addition to this, access to an OHT had important benefits to working with residents with high care needs including dementia and those requiring additional strategies and advice to managing their oral care needs.

*I think the best thing has been the individualised plans. Like a couple of our residents have got quite advanced dementia and one in particular still has her own teeth. So it was really, really difficult to maintain her oral hygiene. But now they just use, not sure what it is, but something special that is more or less like an antibacterial thing. So, we use that for her rather than trying to get a toothbrush and toothpaste into her mouth which just distresses her, distresses the staff because it is distressing her. So I think that is working well.* IDI Facility 6

Many also felt that previously there was not much confidence in how oral health was being attended to. Access to an OHT assisted in improving confidence in staff practice and ability to manage any challenging oral health concerns that were observed.

*I think we flew by the seat of our pants as far as oral health assessments go and it was more or less just going through just the piece of paper, the assessment, the oral health assessment, doing what it said, not really knowing what you were looking for.* IDI Facility 6

The program design also meant that access to incidental hands-on training became more frequent. That is, many respondents commented that the practical observations of the OHT attending to residents was a great training opportunity.

*The oral health therapist was great, we went through everyone’s teeth and it was good because I got to go in there to see the whole business… Yeah, so it was good… And we went through everyone thoroughly. Like their whole health perspective and what could make their oral health worse, yep, I was able to pass that on to others… It was great for me because it was something new.* IDI Facility 7

#### Accessing oral health service

Along with improved awareness of oral health needs and support for managing residents, the model also meant several improvements such as facilitation of communication between the aged care facility and the external oral health service. Due to this improvement in communication, many felt that accessing and organising appointments with the oral health service became more streamlined. Residents were being attended to in a preventative model and, if there were any concerns, it was easy to facilitate a review with the OHT. The access to a tele-dentistry appointment also meant improved communication of treatment and intervention needs with the Dentist, as staff were often present and able to get direct advice from the Dentist; as one respondent advised.

*The tele-dentistry appointment is really good. If (the OHT) meets a problems which needs further suggestion, you know advice, it is easy to ask the Dentist on the spot. And as the representative from here I listen. Therefore, there is less time needed to communicate between staff. You are just there... You can’t do that if you have got to travel to the dental clinic… hopping in the car or arranging a taxi for transporting. It is time consuming and it actually disrupts the residents so this way is great*. IDI Facility 8

#### Implications for residents with high care needs

The benefits for having access to tele-dentistry had enormous impact on minimising need to attend an oral health service. There was no travel or waiting room time required for residents or aged care facility staff member, and no cost for transportation and staffing requirements.

*Yeah because (travel to the oral health service) it is a cost. But if you can use this system (integrated oral health model) that is going to make it easier, and the Dentist is there, like he is visually with you. It is amazing really*. IDI Facility 7

This was particularly important for residents with high care needs including dementia.

*The other good thing about it is we don’t really have to arrange transport…You save time from staffing point of view, it saves, um behavioural problems from a resident point of view... And well this (the aged care facility) is a familiar place. They just go down stairs with familiar people like us… Um what else, it is comfort thing, for everyone involved really*. IDI Facility 8

#### Potential barriers observed

Although mostly positive feedback was reported, there were a few issues that were raised that may pose an issue to implementing the integrated model. This included not enough time for dedicated oral health staff within the aged care facilities to focus on management of the program; consideration that some facilities may not be equipped to take on telehealth technology; access to telehealth technology not in a well-planned and accessible space for residents; and delays in procurement of recommended oral health equipment such as newly recommended toothpaste or changes to toothbrushes. It was also advised that the visiting OHT needed to be skilled in working with residents with dementia and other high care needs.

## Discussion

This study aimed to examine the impact and experience of implementing an integrated oral health program incorporating OHTs and Tele-dentistry in aged care facilities in rural and regional settings. Results show that access to an integrated oral health program has several potential benefits including: assisting performance on meeting accreditation standards; improving access to oral health education for aged care staff and preventative health care for residents; and minimizing disruption to residents, especially those with high care needs such as dementia. Although not able to replicate the full impact of face to face examinations with a Dentist, the integrated model helped to address inequality in access as well as improve oral health education, promotion, disease prevention and timely intervention.

Access to tele-dentistry allowed for referrals to a dental consultant to support locally-based treatment, potentially reducing waiting lists and unnecessary travel. Being seen by a Dentist via the tele-dentistry appointment also enabled residents to remain in a familiar environment; supported by familiar staff including the OHT; and consequently, decreasing the potential for distress of the resident. This was of importance to residents with severe impairments such as dementia who are known to have poorer oral health and often exhibit resistant or uncooperative behaviour or agitation during helping interactions such as oral health [50–53]. Those with severe impartments are also likely to have other complications that would impact on participating in an oral health appointment such as ability to transfer to a Dentist’s chair. This was alleviated by residents being able to be seen in equipment suitable to their needs, in the comfort of their own room. The real-time communication between Dentist, OHT and aged care staff member also facilitated oral health education and training as well as addressing the individual needs of the resident.

Access to tele-dentistry alone was not observed to solely contribute to the improvement of oral health management within the aged care facilities. A combination of improved communication and integration with an oral health service and involvement of an OHT was also important. Aged care staff perceived they had limited skills to be able to provide comprehensive oral health and that someone with oral health training should be involved. Although there is scope for aged care staff to be involved in oral health care and intervention, the qualitative findings indicate that other competing demands within the facilities often took precedent. The reduced confidence in ability to provide specific oral health measures to residents may be attributed to lack of experience due to lack of time as well as the additional impact of working as a generalist in a rural facility. This may in turn be affected by reduced access to training and peer support particularly for those in multi-purpose health centres or more isolated towns.

The use of an OHT in aged care facilities has already been shown to have a significant impact on improving oral health of residents such as plaque scores [28] and being able to appropriately refer on to a Dentist for further care [29]. In our study, the visiting OHT was reported to improve teaching and learning opportunities for oral health, and access to preventative rather than reactive services. Increased access to training and education in oral care by RACF staff has been shown to improve or maintain the oral health of residents, particularly those with initial poor oral hygiene [54–56]. However, there is also evidence that training of RACF staff alone does not always solve the problem [25, 57]. Therefore, the ongoing access to the OHT for ongoing training (even incidental) is important; particularly in light of high turnover of staff in some RACFs [26]. This research also suggested that the integrated oral health program improved visibility of oral health in the RACF which assisted in increasing priority of oral health among RACF staff, a finding which confirms expectations of other studies [58].

The interplay of contextual issues involved in improving oral health in the residential aged care setting are highly complex. There are many interdependent factors across organizational, professional and sectorial boundaries [59]. This study demonstrates the effectiveness of a collaborative approach to managing oral health in RACFs between the oral health service, RACF and tele-health team. A multidisciplinary partnership model to oral health in RACFs has shown high potential for reducing access barriers for residents in RACFs and improving oral health outcomes in a sustainable manner [60, 61]. RACFs, particularly in rural areas, require oral health services to consider them as part of the overall oral health of the community. This confirms previous studies that show that without specialist programs in the RACF, residents may not receive routine oral health treatment and dental care is mostly demanded in response to a problem [50]. This integrated model incorporating OHTs and tele-dentistry provides a potentially efficient model that allows for improved relationship across services.

### Limitations

Residents that participated in the oral health quality of life survey were limited to those without major cognitive impairment and with sufficient communication skills to complete the questionnaire. The small sample size obtained for this survey is reflective of the large population of residents with dementia and other cognitive impairment at the study sites. Results from the quality of life survey are therefore not generalizable to residents with dementia. Those with dementia and communication difficulties are also likely to not be able to communicate oral health concerns and this may therefore impact negatively on their oral health quality of life. Being able to communicate oral health need to staff may have a positive impact on oral health quality of life and therefore results of the survey may show a more positive situation than what is experienced.

This research was particularly looking at the impact and experience of the integrated program in aged care facilities. The facilities that were included in the group that did not have integrated program did range from being in communities with no local Dentist or oral health support to communities where there was an active private and/or public oral health service. The impact of not having any support is likely to have major implications for successful oral health program. As previous research has alluded to, a multidisciplinary approach to oral health is likely to be more effective and sustainable. The contribution of a supportive and accessible local oral health service for the aged care facilities has not been accounted for in this study.

## Conclusions

Oral health problems in aged care facilities are a result of barriers to oral health services, difficulties of managing oral health of high care residents, and poor skills and lack of time of facility staff. The integrated oral health program incorporating OHTs and tele-dentistry shows potential to improve the oral health outcomes of residents of aged care facilities. The benefits were also observed to be of importance to high care residents including those with dementia and other significant cognitive and physical disabilities. Facilities without easy access to an oral health service will also likely benefit due to the increased support and training opportunity that the program offers.

## Additional file

Additional file 1: Interview guide for focus group discussions and in-depth interviews. (DOCX 14 kb)

## Abbreviations

GOHAI: Geriatric Oral Health Assessment Index
OH: Oral Health
OHQoL: Oral Health Quality of Life
OHT: Oral Health Therapist
RACF: Residential Aged Care Facility
