# Supplementary material for: An integrated oral health program for rural residential aged care facilities: a mixed methods comparative study
Source: BMC Health Serv Res. 2018 Jul 3;18:515. doi: 10.1186/s12913-018-3321-5 (PMC6029389; doi:10.1186/s12913-018-3321-5)
Supplement: Supplementary file 1 — Interview guide for focus group discussions and in-depth interviews. (DOCX 14 kb) [file 12913_2018_3321_MOESM1_ESM.docx]

**Focus Group Discussion and In-depth interview Guide**

**Experience of oral health care in RACF**

Experience with managing oral health care needs of residents in RACF

- Describe the current oral health service within the RACF.
- What has been the experience of the current oral health program/ integrated oral health program?
- Perceptions of barriers/challenges and facilitators to improving oral health for resident?
  - - What worked and what didn’t?
    - What are the gaps and limitations?
- Describe any impact the current program has for residents.
- Describe any impact that the program has had for staff.

Perceptions of oral health care needs in RACF

- What are the oral health care needs of this population?
- Who are/ what groups are being most impacted/ should be targeted?
- How serious is the problem of these identified needs?

**Recommendations**

- What are the perceived actions that should be taken?
- What recommendations for the future do you have?
